# Optical properties of the jellyfish surface above the waterline: microvillar array in pleustonic hydrozoans

**DOI:** 10.1186/s40851-025-00253-4

**Published:** 2025-09-02

**Authors:** Euichi Hirose, Nicolò Brunelli, Daisuke Sakai, Hiroshi Kakiuchida, Jun Nishikawa

**Affiliations:** 1 Faculty of Science, University of the Ryukyus, Nishihara, Okinawa 903-0213 Japan; 2 Department of Biology, University of Padova, Padova, Italy; 3 School of Regional Innovation and Social Design Engineering, Kitami Institute of Technology, Kitami, Hokkaido Japan; 4 Innovative Functional Materials Research Institute, National Institute of Advanced Industrial Science and Technology (AIST), Nagoya, Aichi Japan; 5 Department of Marine Biology, School of Marine Science and Technology, Tokai University, Shimizu, Shizuoka Japan

**Keywords:** Anti-reflection, Microvillar array, Pleuston, Pneumatophore, Refractometry, Rigorous coupled wave analysis (RCWA)

## Abstract

**Background:**

The transparent jellyfish body is often difficult to see underwater, as its refractive index is similar to that of seawater, resulting in a low light reflectance on the body surface. Nevertheless, the outlines of jellyfish can be recognized by the slight reflection of light from their body surfaces. In some jellyfish species, the epidermis covering the body surface has an array of microvilli, nanostructures that can potentially reduce light reflection. However, the anti-reflective effect is minimal in water, as the difference in the refractive indices of tissue and seawater is so small that reflectance is low, even on flat surfaces. In jellyfish that have pneumatophores, structures used in floating and drifting on the sea surface, light reflection on the surface is expected to be large and noticeable owing to the large differences in refractive indices between the pneumatophore exposed above the water surface and air. In the current study, we examined the epidermal ultrastructure and refractive index of the pneumatophores of a Portuguese man o’ war (*Physalia physalis*) and a by-the-wind sailor (*Velella velella*).

**Results:**

The refractive index of *P. physalis* pneumatophores measured with an Abbe refractometer was approximately 1.344. Microvillar arrays were found in epidermal cells of both *P. physalis* and *V. velella*. Based on the length, thickness, and pitch of the microvilli, we constructed simplified structural models for the simulation of light reflection using rigorous coupled wave analysis (RCWA). Our simulations showed that reflectance on the microvillar models could be greater or less than that on the flat surface, depending on light conditions (wavelength and angle of incidence), but with an overall effect of reduced reflection. Reflection reduction in microvillar models was particularly significant at large incident angles, where reflectance was extremely high on the flat surface.

**Conclusions:**

Microvillar arrays found on the epidermis potentially reduce surface reflections of the pneumatophore and contribute to the reduction in visibility of the pleustonic hydrozoans above the sea surface. Moreover, less reflection at the pneumatophore surface indicates greater transmission of light through transparent bodies, potentially providing a counter-illumination effect that obscures the shadow of the hydrozoan bodies, depending on the intensity of ambient light.

**Supplementary Information:**

The online version contains supplementary material available at 10.1186/s40851-025-00253-4.

## Background

**Fig. 1:**
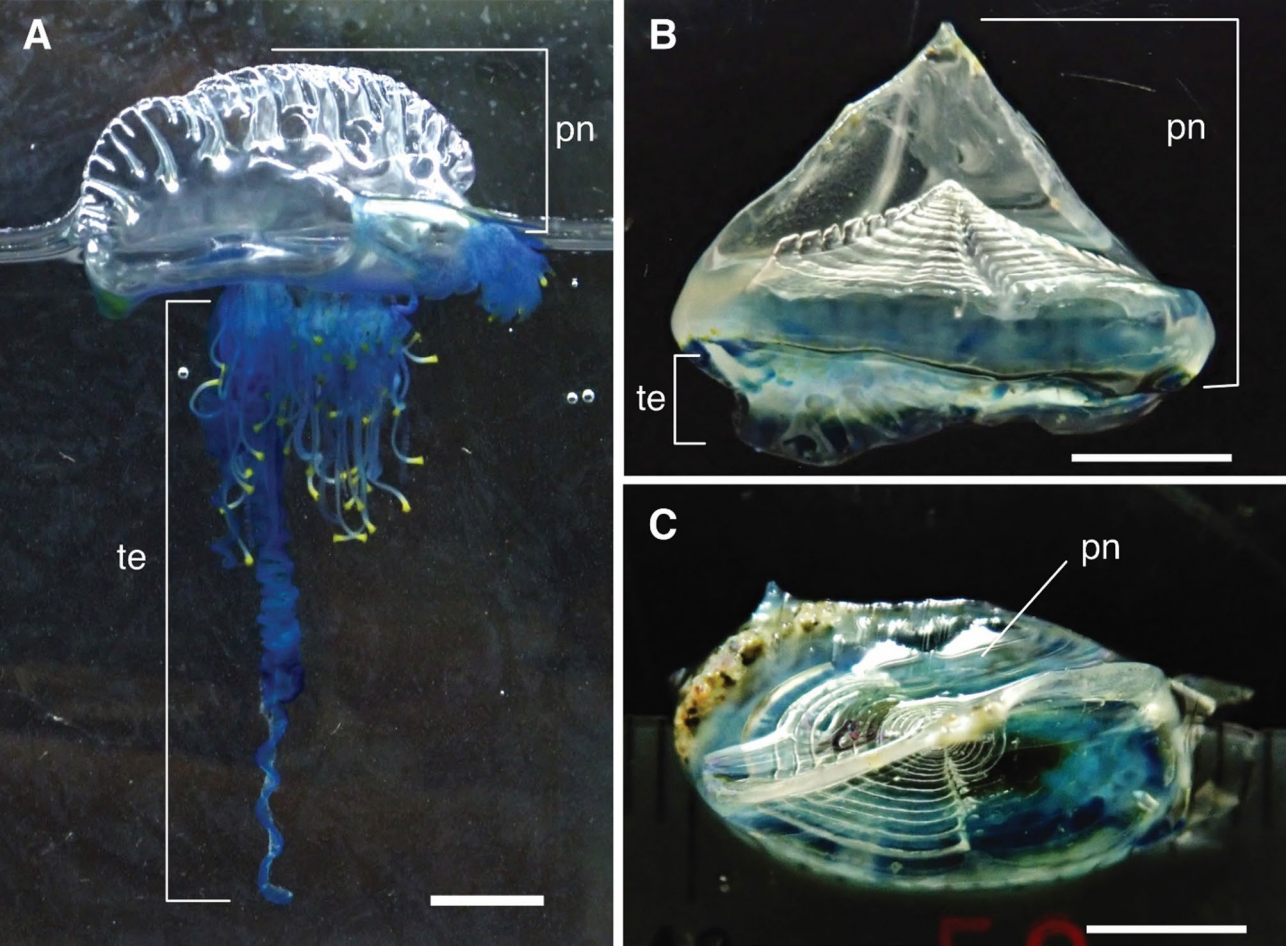
Live specimens of *Physalia physalis* (**A**) and *Velella velella* (**B**, **C**). Lateral view (**A** and **B**) and Upper view (**C**). pn, pneumatophore; te, tentacles. Scale bars: 10 mm in **A**, 5 mm in **B** and **C**

Modern warships are sometimes painted in camouflage patterns, but what about the Portuguese man o’ war as it drifts silently on the sea surface? *Physalia physalis* (Linnaeus, 1758) (Hydrozoa: Siphonophora: Phisaliidae) is a colonial hydrozoan that is widely known as a dangerous pleuston owing to its venomous nematocysts, which occasionally cause fatal accidents (Fig. 1A). The *P. physalis* colony floats on the sea surface with a gas-filled float (pneumatophore), the distinctive shape of which is the origin of the common name for this species. *Velella velella* (Linnaeus, 1758) (Hydrozoa: Anthoathecata: Porpitidae), known as by-the-wind sailor, is another colonial hydrozoan, and it also has a gas-filled pneumatophore (Fig. 1B, C). Both species are carnivorous, and their bluish tentacular zooids, each bearing numerous nematocysts, extend into the water to capture prey.

In our ultrastructural survey of exumbrellar surfaces for nanostructures that suppress visibility in cnidarian medusae, we found that the exumbrellar epidermis of some species had arrays of microvilli, which may reduce surface light reflection; moreover, optical simulations showed that the microvillar array may reduce surface light reflection [1]. Although such microvillar arrays are slightly larger than the moth-eye structure (nipple array), which is widely known as the non-reflective structure in insects [2, 3], the results of our optical simulations suggested that microvilli reduce light reflection on the cnidarian exumbrellar surface [1], similar to the effect of nanoscale nipple arrays on the tunic surface in some tunicates [4–7]. However, surface reflections are relatively small in jellyfish, even without surface nanostructures, because the difference in the refractive index between jellyfish tissue and seawater is very small. Therefore, the anti-reflective effect of the microvillar array in water is much smaller than that of the moth-eye structure of insects in air. The greater the difference in the refractive index between a surface tissue and its environment, the greater the light reflectance. Thus, insects living in an aerobic regime with a low refractive index (ca. 1) have a greater reflectance at their body surface, whereas jellyfish in an aquatic environment with a high refractive index (> 1.33) exhibit a smaller reflectance. This raised the question, would jellyfish possessing tissue exposed to air exhibit anti-glare structures?

The pneumatophores of *P. physalis* and *V. velella* are mostly exposed to air. In the present study, we measured the refractive index of the pneumatophore surface of *P. physalis* and describe fine structures of the epidermis in *P. physalis* and *V. velella*. Based on these observations, we carried out rigorous coupled wave analysis simulating light reflectance on pneumatophore surface models to estimate the difference in reflectance with and without surface nanostructures.

## Methodology

### Animals

Two *P. physalis* with pneumatophore lengths of 2.2 and 3.0 cm along the long axis and two *V. velella* with pneumatophore lengths of 1.6 and 1.8 cm along the long axis were collected by some members of Tokai University Lifesaving Club LOCO at Sagara Sun Beach, Sagara, Makinohara, Shizuoka, Japan (34.682194 N, 138.205000E) on 6 August 2022. All specimens were fixed with 2.5% glutaraldehyde in 0.45 M sucrose and 0.1 M cacodylate (pH 7.4) and stored at 4 °C for microscopy.

Two additional *P. physalis* that had washed up along the Miho coast, Shimizu, Shizuoka, Japan (34.9863167 N, 138.5168809E) were collected by hand on 24 July 2024. The pneumatophores were approximately 3.2 and 5.9 cm along the long axis. These live specimens were immediately brought to the laboratory for the measurement of the refractive indices.

### Measurement of refractive index

Pneumatophores of two *P. physalis* specimens were cut into sheets and the surface refractive index was measured using an Abbe refractometer (DR- A1-Plus, Atago Inc.) at 22.8 °C with 589-nm light (D line). The measurement accuracy of the refractometer was ± 0.0002 according to the manufacturer’s specifications. Measurements were performed in triplicates for each specimen. We were unable to obtain *V. velella* specimens for measuring the refractive index.

### Microscopy

The pneumatophores were cut into pieces of approximately 5 mm square in the fixative. Following a brief rinse with 0.1 M cacodylate and 0.45 M sucrose, the specimens were post-fixed in 1% osmium tetroxide in 0.1 M cacodylate for 1.5–2 h on ice. They were then dehydrated through an ethanol series, cleared with *n*-butyl glycidyl ether, and embedded in epoxy resin. Sections of approximately 0.1 μm thickness were stained with uranyl acetate and lead citrate and examined under a transmission electron microscope (TEM: JEM-1011, JEOL) at 80 kV. Electron micrographs were captured using a digital camera (Orius SC1000, Gatan). ImageJ 2.1.0 was used to assess the dimensions of the structures. Surface structure models for the reflectance simulations were constructed based on the average values of the measured dimensions.

### Simulation of light reflectance

Light reflection was calculated at the border between air and the models for pneumatophore surfaces with and without microvillar arrays (i.e., structured and flat surfaces) with rigorous coupled wave analysis (RCWA) using DiffractMOD software (Synopsys, Inc., Sunnyvale, CA, USA). The difference in refractive indices (Δ*n*) between air and the tissue surface was assumed to be 0.344, according to measurements with a refractometer in *P. physalis*. In this simulation, we used polarized light: transverse electric waves (TE waves) and transverse magnetic waves (TM waves). The parameters for the simulation included the wavelength of light (λ: 280–1000 nm, every 1 nm) and angle of incidence of light (*θ*: 0–89.9°, every 0.1°).

## Results

### Refractive index of pneumatophore surface

Refractive indices were measured for two *P. physalis* specimens with pneumatophore lengths of 3.2 and 5.9 cm along the long axis. The indices were 1.3428, 1.3459, and 1.3433 for the former specimen (average, 1.3440) and 1.3439, 1.3443, and 1. 3440 for the latter specimen (average, 1.3441).

### Ultrastructure

**Fig. 2:**
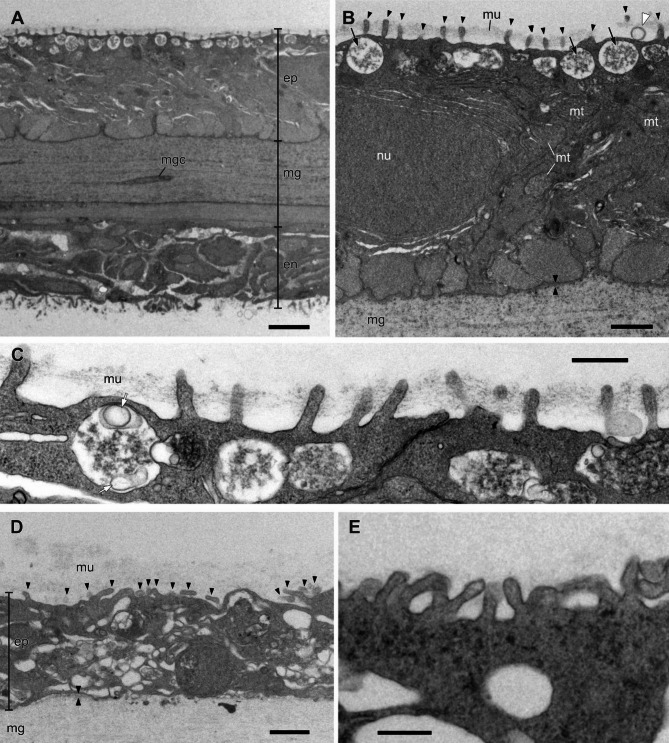
Transmission electron micrographs of the pneumatophore epidermis of *Physalia physalis* (**A**–**C**) and *Velella velella* (**D**, **E**). **A**, Integumentary tissue of pneumatophore. **B**, Enlargement of the epidermis. **C**, Microvilli on the apical side of the epidermis. **D**, Epidermis. **E**, Enlargement of the microvilli. Arrowheads, microvilli; arrows, round vesicles beneath the apical membrane; facing arrowheads indicate basal lamina; white arrowheads, vesicle on the apical membrane; white arrow, small vesicles in the round vesicle. ep, epidermis; en, endoderm; mg, mesoglea; mgc, mesoglea cell; mt, mitochondrion; nu, nucleus; mu, mucus layer. Scale bars: 10 μm in **A**, 1 μm in **B** and **D**, 0.5 μm in **C** and **E**

In *P. physalis*, the outermost tissue of the pneumatophore comprises a thin mesoglea (ca. 15 μm thick) sandwiched between the epidermis (ca. 25 μm thick) and endoderm (ca. 20 μm thick) (Fig. 2A). The mesoglea layer is moderately electron-dense and consists of fibrous material. Fusiform cells are occasionally observed within this layer. The epidermis and endoderm are composed of cuboidal epithelia.

In the epidermal cells of *P. physalis*, microvilli emerge regularly from the apical membrane (arrowheads in Fig. 2B). The microvilli tend to extend straight outward, roughly perpendicular to the apical membrane. Fine fibrous material forms a mucus layer that loosely fills the space between the microvilli (Fig. 2B, C). A clear vesicle was occasionally observed on the apical membrane (white arrowhead in Fig. 2B). Round vesicles are arranged in a row beneath the apical membrane and contain fibrous material of moderate electron density (arrows in Fig. 2B). The basal membrane is uneven, and the basal lamina is a loose, fibrous layer of approximately 0.01 μm in thickness.

In *V. velella*, a mesoglea formed from a fibrous matrix is overlaid with an epidermis of 2–6 μm thickness, and a mucus layer of various thicknesses covers the epidermal surface (Fig. 2D). While microvilli emerge from the apical membrane of the epidermis (arrowheads in Fig. 2D), many are irregularly flexed, while a few extend straight (Fig. 2E). The cytoplasm contains many clear vesicles of varying sizes. The basal membrane is uneven, and often projects short cytoplasmic processes in the mesoglea. The basal lamina is a loose, fibrous layer of approximately 0.01 μm thickness (Fig. 2D).

**Fig. 3:**
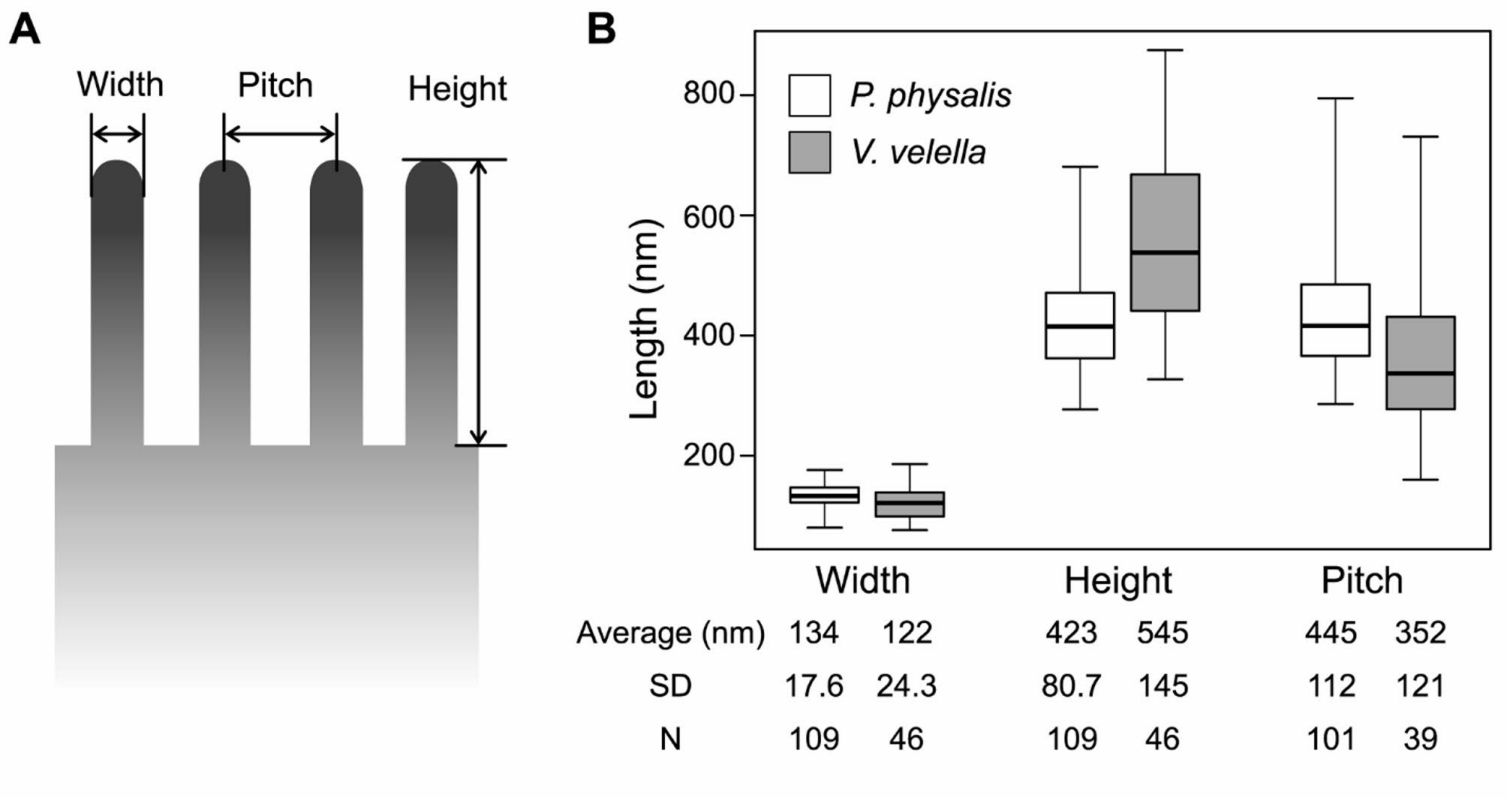
Dimensions of the arrays of microvilli in *Physalia physalis* and *Velella velella.*** A**, Schematic diagram of microvilli showing the measurement points. **B**, Box plot of the measured values with averages, standard deviations (SD), and number of measurements (N)

The dimensions of the array of microvilli were investigated for the *P. physalis* specimen with a 2.2-cm pneumatophore and *V. velella* specimen with a 1.8-cm pneumatophore. The width, height, and pitch of the microvilli were measured in five thin sections for each specimen (Fig. 3A). Measurements were not possible in areas where the microvilli were cut obliquely; thus, serially arranged microvilli were selected from each section for measurement. Whereas the epidermal microvilli were irregularly flexed in *V. velella*, the length of microvilli was measured as height. The dimensions of the microvillar array are summarized in Fig. 3B. The dimensions of the microvillar arrays were not strictly uniform and some variations were observed between the thin sections used for these measurements (Supplemental Fig. S1).

### Simulations of light reflectance

**Fig. 4:**
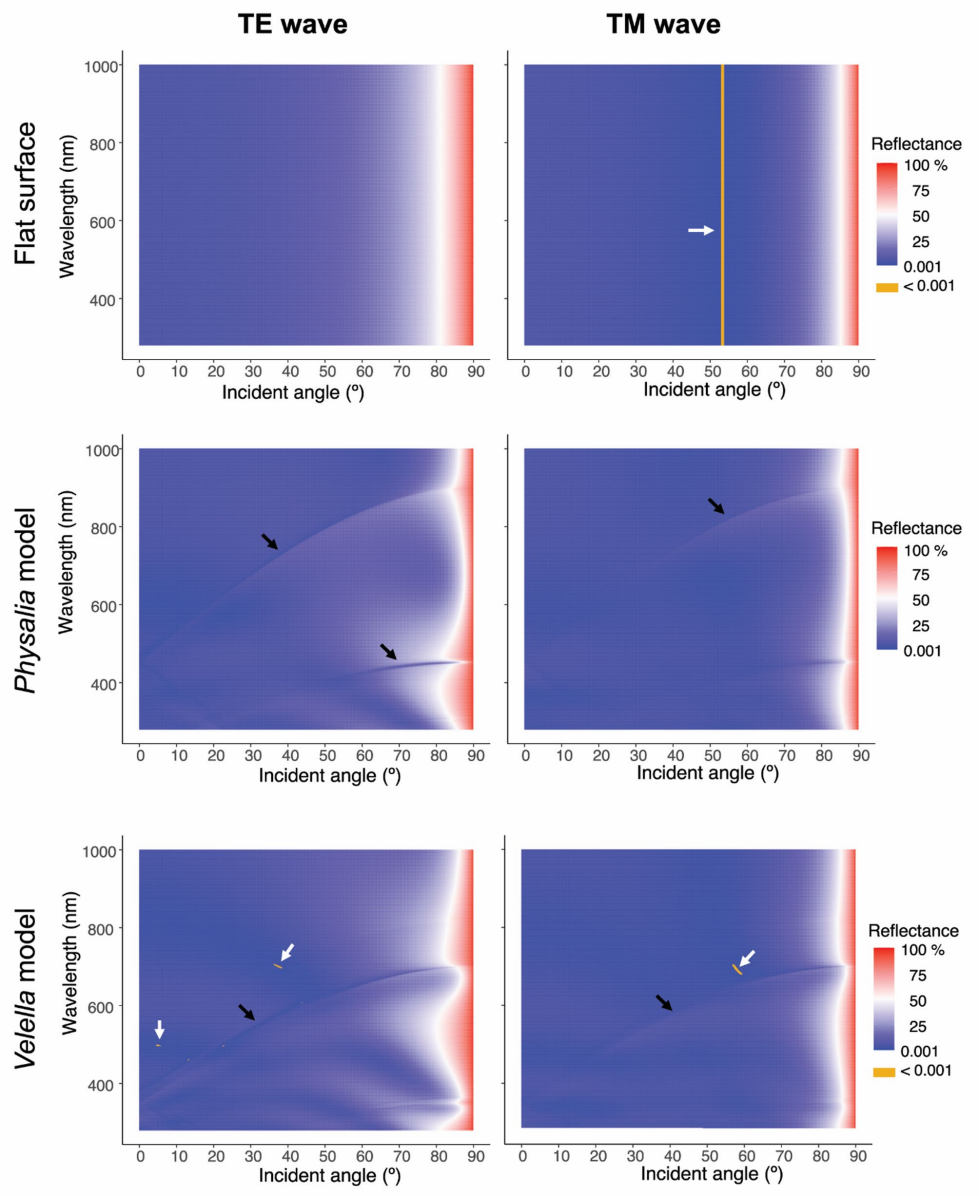
Heat maps of light reflectance on the unstructured (= flat) model (top), *Physalia* model (middle), and *Velella* model (bottom) for the TE waves (left column) and TM waves (right column). Black arrows indicate curvilinear conditions in which the reflectance was lower (or higher) than the surrounding area on the nanostructured surfaces. Orange areas indicated by white arrows indicate less than 0.001% reflectance

Simplified surface models were constructed based on the average dimensions of *P. physalis* and *V. velella* microvillar arrays to simulate light reflection. In these models, the microvilli were represented as evenly spaced cylinders with hemispherical tips; the width, height, and pitch of the modeled microvilli were 130, 420, and 450 nm for *P. physalis* and 120, 550, and 350 nm for *V. velella*, respectively. Because the refractive index of the *P. physalis* pneumatophore was approximately 1.344 in our refractometric measurements, in this simulation we assumed the difference between the refractive indices of air and the pneumatophore surface to be 0.344 in the simulation. This value was applied to both the *P. physalis* and the *V. velella* models, as well as to the flat surface model. Using RCWA, we calculated the reflectance of TE and TM waves corresponding to incident angles and wavelengths and drew heat maps consisting of 648,900 cells for each (Fig. 4).

On the flat surface, the light reflectance in percentage of TE waves was approximately 2% at an angle of incidence of 0° and increased as the incident angle increased, irrespective of wavelength, including ultraviolet (280–400 nm) and near-infrared (800–1000 nm) spectra (top left in Fig. 4). Reflectance increased rapidly when the angle exceeded 60°; the reflectance was 25% at 71.5°, 50% at 81°, and > 90% at 88.7°. The light reflectance of TM waves was approximately 2% at an angle of incidence of 0°, as found for the TE wave, and the reflectance of the TM wave was minimum (< 0.0002%) at 53.3°, due to Brewster’s angle, and rapidly increased with the increase in incident angle (top right in Fig. 4); the reflectance was 25% at 80.3°, 50% at 85.1°, and > 90% at 89.3°. The reflectance was < 0.001% within the orange area in the heat map for the TM wave on the flat surface (white arrows in Fig. 4).

In the nanostructure model of *P. physalis*, the light reflectance of TE waves tended to increase with increasing incident angle as found on the flat surface, but the change varied with wavelength (middle-left in Fig. 4). In addition, conditions where the reflectance was lower (or higher) than that of the surrounding area were observed on the heat map in the form of curves (black arrows in Fig. 4). For instance, the reflectance of 600-nm light was 0.055% at 0º, 25% at 83.2°, 50% at 87º, and > 90% at 89.6°, while the minimum reflectance was 0.016% at 10º. The reflectance of 800 nm light was approximately 2% at 0º, 25% at 81.4°, 50% at 86.5°, and > 90% at 89.5°, while the minimum reflectance was 0.48% at 51º. Similar curves were also observed in the heat map for the TM waves (middle right in Fig. 4). The reflectance of the TM waves decreased around Brewster’s angle, as found on the flat surface, but the decrease was remarkably less. The minimum reflectance of the TM wave was 0.91% at 51° for 400 nm light, 0.28% at 53–53.6° for 600 nm light, and 0.91% at 51° for 800 nm light. The light reflection of the nanostructure model based on *V. velella* (bottom in Fig. 4) was roughly similar to that of the *Physalia* model. In the heat maps, conditions with higher (or lower) reflectance than their surroundings appear as curves, but the locations of the curves in the *V. velella* model were different from that in the *Physalia* model. Similar to the *Physalia* model, the decrease in TM wave reflection around the Brewster angle was also less prominent.

**Fig. 5:**
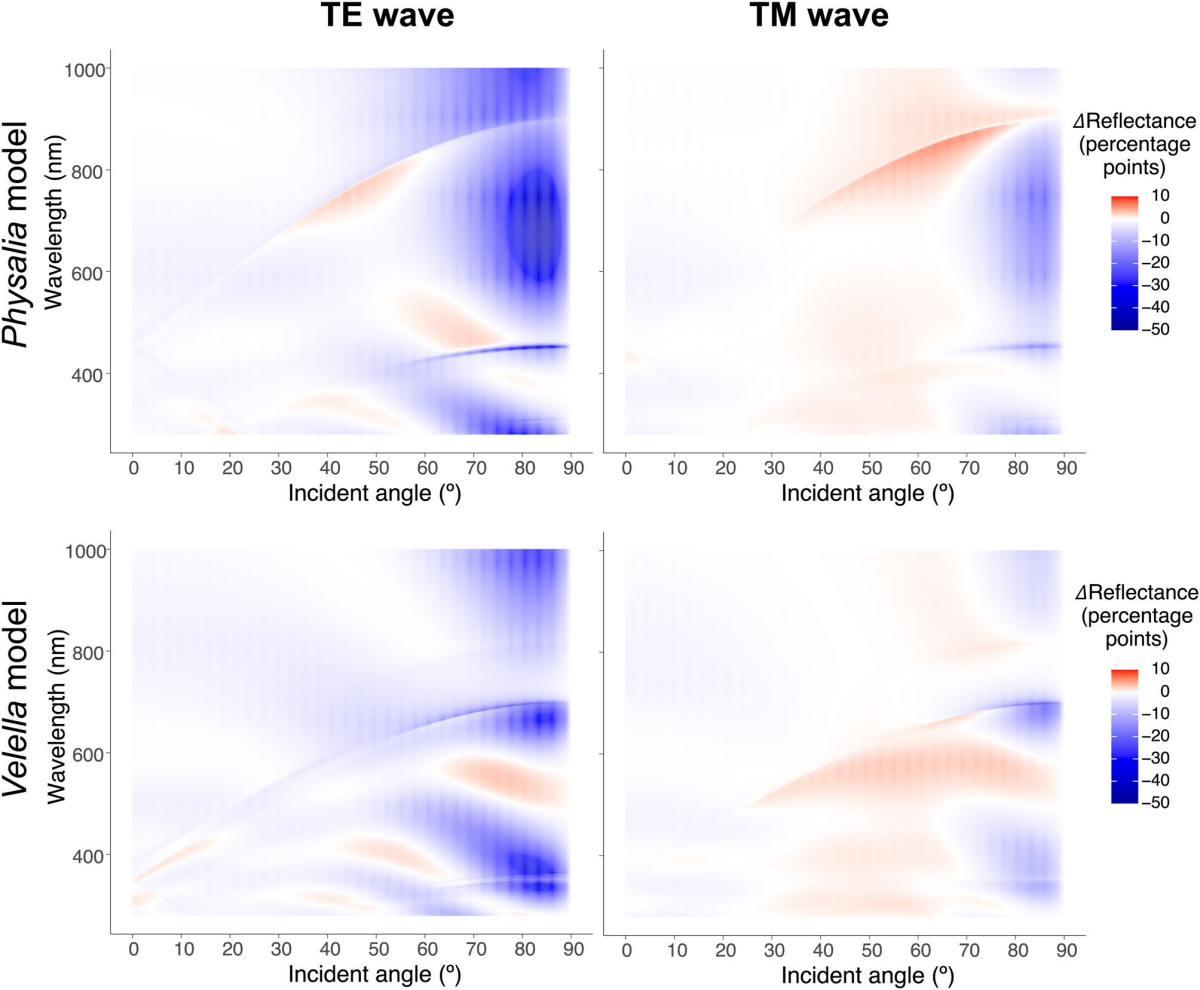
Heat maps showing differences in light reflectance between nanostructured (*Physalia* model and *Velella* model) and unstructured (= flat) surfaces. The reflectance of the nanostructured surface was greater than that of the flat surface in the reddish area and smaller in the bluish area

The heat maps in Fig. 5 show differences in light reflectance between nanostructured (*Physalia* model and *Velella* model) and unstructured (= flat) surfaces; the reflectance (%) was greater on the nanostructured surface than on the flat surface in the reddish area, smaller in the bluish area, and almost the same in the whitish area. Overall, the reflectance of the nanostructures was lower than on the flat surfaces in the TE wave, occupying approximately 92% (*Physalia* model) and 94% (*Velella* model) of the area on the heat map, respectively. Even in the range of incidence angles above 80º, where the reflectance is approximately 50% or higher on the flat surface, the reflectance is lower than that at the flat surface in areas of approximately 97% of the *Physalia* model and 89% of the *Velella* model surfaces, respectively. Notably, for light of wavelength 600–800 nm, the reflectance of the *Physalia* model was more than 10 percentage points lower than that of flat surfaces in the range of approximately 80º to 88º. Moreover, in the range of incident angles greater than 80º, 22.3% of the areas had reflectance more than 30 points lower, and 5.6% had reflectance more than 40 points lower in this heat map (upper left in Fig. 5). The reflectance of TM waves on the nanostructures was smaller than that on the flat surface in the areas of approximately 57% (*Physalia* model) and 62% (*Velella* model), respectively, in the heat maps (left column in Fig. 5). As described above, the reflectivity of TM waves around Brewster’s angle is significantly smaller on flat surfaces; however, this decrease in reflectivity is much less prominent on nanostructured surfaces (right column in Fig. 4). Therefore, the reflectivity of the nanostructure is greater than that of the flat surface in the range of incidence angles from approximately 30º to 70º, including Brewster’s angle (right column in Fig. 5). In contrast, in the range of incidence angles above 80º, where the reflectance of TM waves is approximately 25% or higher on the flat surface, the reflectance is lower than the flat surface in the areas of approximately 89% (*Physalia* model) and 85% (*Velella* model), respectively.

## Discussion

In the two pleustonic hydrozoans *P. physalis* and *V. velella*, arrays of microvilli regularly emerged from the apical membrane of the epidermis. Similar microvillar arrays have been reported from the exumbrellar epidermis of cubozoan jellyfish, such as *Chironex yamaguchii* Lewis and Bentlage, 2009, and optical simulation using RCWA suggested that these nanostructures potentially reduced light reflectance at the exumbrellar surface [1]. Interestingly, the dimensions of the microvilli of *C. yamaguchii* (approximately 500 nm in height and 365 nm in pitch) are not significantly different from those of *P. physalis* (approximately 420 nm in height and 450 nm in pitch). The scyphozoan jellyfish, *Mastigias papua*, also has dense microvilli on its exumbrellar epidermis, but their height exceeds 2 μm [1]. However, the optical properties imparted by surface nanostructures have very different functional significance for exumbrellas in seawater and pneumatophores exposed to air. Because the difference in refractive index between umbrella tissue and seawater is very small (≈ 0.001), the surface reflectance of unstructured surfaces is also quite small; thus, any nanostructural effect in reducing reflectance is presumed to be limited. However, the difference in refractive index between air (ca. 1) and pneumatophore (ca. 1.344) is much greater than that between seawater (ca. 1.343) and tissue, resulting in greater reflectance of the pneumatophore in the air, and thus the effect of the nanostructure in reflectance reduction is expected to be much more pronounced in the air. A loose mucus-like structure was present on the surface of the epidermis, where the microvilli were arrayed (Fig. 2B–D). This mucus layer may serve as an optical layer on the surface, and the measured refractive indices reflect the optical properties of the mucus layer. The mucus layer is also important for protecting the epidermis from drying out from exposure to air, and the microvillar array may effectively retain mucus on the surface.

In the present study, we performed simulations of light reflection using RCWA and compared the reflectance on a flat surface and simplified nanostructure models based on the ultrastructure of *P. physalis* and *V. velella*. In the heat maps of reflectance on the nanostructure models, reflectance was lower (or higher) than the surrounding area under different conditions (wavelength–incident angle), which when plotted took the form of curves in the heat maps (black arrows in Fig. 4). The conditions that make up these curves may be due to diffracted light, as the nanostructures in the models were spaced at strictly equal intervals. Because the pitch of the microvillar arrays of jellyfish tissue is not strictly uniform (Fig. 2, Supplemental Figure S1), the diffraction effect is not expected to be as pronounced in actual animal tissue.

In the heat maps showing the differences in reflectance between the nanostructured models and flat surfaces (Fig. 5), the reflectance of the nanostructured models was mostly smaller than that of the flat surface. In particular, the reflectance of the TE wave on the *Physalia* model was markedly reduced in the range of large incident angles (e.g., >80º), at which the reflectance on the flat surface was 50% or more, and the flat surface had a TE wave reflectance up to 43.3 percentage points greater than that of the *Physalia* model (upper left in Fig. 5). The considerable reduction in reflectivity due to the nanostructures suggests that the microvillar array of the pneumatophores makes the pleustonic hydrozoa less visible from the air. The reflectance was larger in the nanostructured models, depending on the wavelength and incident angle. A major case is the reflectance of the TM wave around Brewster’s angle of incidence (right column in Fig. 5). The reflectance was extremely small (< 0.0002%) at Brewster’s angle on a flat surface; however, the decrease in reflectance around Brewster’s angle was not prominent in the nanostructured models. Under the conditions where the reflectance of the TM wave on the nanostructured models was higher than that of the flat surface, the maximum difference in reflectance was only 7.1 percentage points for the *Physalia* model and 3.2 percentage points for the *Velella* model. Accordingly, the effects of the reflectance increase due to the nanostructures are considered to be very small compared to those of the reflectance decrease due to the nanostructures at larger incident angles, at which the reflectance of the flat surface was greater.

What are the benefits to hydrozoans in reducing light reflection on their float surfaces? Some seabirds are known to prey on pleustonic jellyfish, including *Physalia* and *Velella*, which have strongly venomous cnidocytes [8, 9]. Because seabirds are visual predators, less reflective floats are expected to be less visible to those predators above the water surface. However, it is uncertain how serious the predation pressure by seabirds is on pleustonic hydrozoans. It is likely that the major predators of *Physalia* and *Velella* are submerged or floating marine animals such as sea turtles, fish, heterobranchs, and cephalopods [8]. Reduced light reflection on the pneumatophore indicates greater transmission of light through transparent bodies, potentially providing a counter-illumination effect that obscures the shadow of the jellyfish during the daytime [10]. Additionally, as *Physalia* and *Velella* are carnivores that capture prey with their tentacles with venomous cnidocytes, being less visible underwater may confer a significant advantage in their ambush hunting behaviors.

**Fig. 6:**
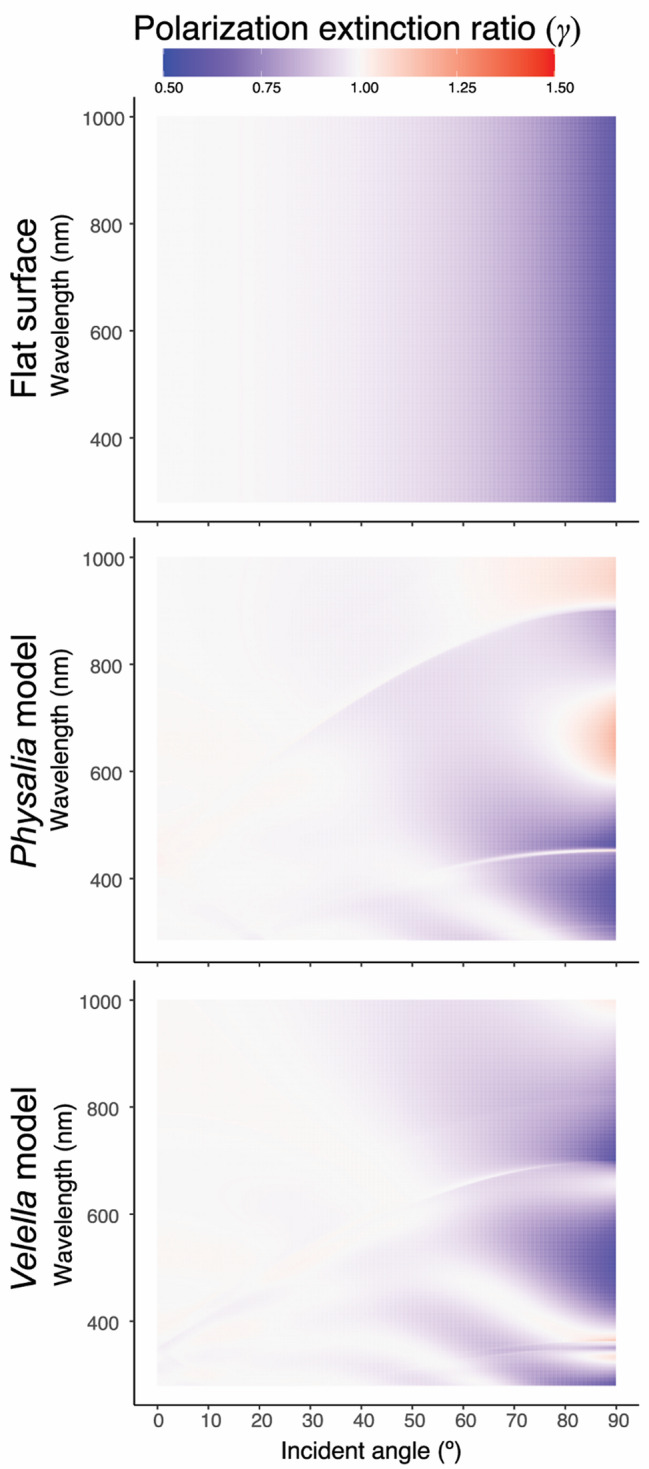
Heat maps of transmittance ratios of TE waves per TM waves (polarization extinction ratio: γ) on flat surface (top), *Physalia* model (middle), and *Velella* model (bottom). Reddish, TE > TM; light gray, TE = TM; bluish; TE < TM

Various invertebrates have a vision system that utilizes polarized light to improve object detection [e.g., 11, 12], which may be effective in visualizing transparent objects, such as jellyfish [13]. We compared polarization extinction ratios through the hydrozoan float into the water based on the reflectivity of the nanostructured and unstructured models (Fig. 6). As the reflectance of the TM wave is extremely small around Brewster’s angle, the reflection of the TM wave is smaller than that of the TE wave at the water surface. Therefore, more TM waves are transmitted into the water column than are TE waves. According to the results of the present simulation, nanostructured models transmit more TE waves than flat models, while reflecting more TM waves around Brewster’s angle. Sunlight transmitted through pleustonic hydrozoans consists of more TE waves and fewer TM waves than does light transmitted through the water surface, which may have the effect of deceiving animals that use polarized light for vision.

As mentioned above, some jellyfish, such as *C. yamaguchi* (Cubozoa) and *M. papua* (Scyphozoa), have dense microvilli in their exumbrella epidermis, but other jellyfish do not, making it difficult to assume a common origin for epidermal microvilli on exumbrellas and hydrozoan pneumatophores. For further discussion, ultrastructural comparisons of the exumbrella and pneumatophore epidermis are required across a wide range of jellyfish species. It has also been pointed out that nanoscale nipple arrays may not only reduce light reflection, but also impart various properties to the surface, such as modification of water wettability [14], anti-contamination [14–16], bubble repellency [17], suppression of cellar function [18], and anti-biofouling [19–21]. The microvillar array of the pneumatophore epidermis in *P. physalis* and *V. velella* may also serve some such functions. The same may also be true for the exumbrellar epidermis in other jellyfish underwater.

## Conclusion

The epidermis of the pneumatophores in the pleustonic hydrozoans *P. physalis* and *V. velella* has a dense array of microvilli, and this nanostructure may modulate the optical properties of the pneumatophore surface. The simulation of light reflection using RCWA indicated that nanostructures reduced reflectance, particularly in the range of higher incident angles. It is presumed that these hydrozoan floats are low-reflective objects above the sea surface and brighter objects when looked up from water. Therefore, the microvillar array may reduce the visibility of these transparent jellyfish bodies during the day. For the TM wave, the light reflection around Brewster’s angle is expected to be greater on the structured surface than on the flat surface. This may have a disguising effect on visual systems using polarized light. Microvillar arrays can serve a variety of functions, such as increasing the surface area of the epidermis and retaining mucus. These structures have been reported in several species of cnidarians [1], and may be independently developed in each taxon for their own functional needs.

## Supplementary Information

Below is the link to the electronic supplementary material.

**Supplementary Material 1: Figure S1.** Variations in microvillar height and pitch among distinct thin sections (I–V). A, *Physalia physalis*. B, *Velella velella*. Numbers in parentheses indicate the number of measurements within each section.

## Data Availability

The datasets supporting the conclusions of this article are included within the article and its additional files.

## Abbreviations

RCWA: rigorous coupled wave analysis
TEM: transmission electron microscope
TE wave: transverse electric wave
TM wave: transverse magnetic wave
