# Supplementary material for: Optical properties of the jellyfish surface above the waterline: microvillar array in pleustonic hydrozoans
Source: Zoological Lett. 2025 Sep 2;11:7. doi: 10.1186/s40851-025-00253-4 (PMC12406395; doi:10.1186/s40851-025-00253-4)
Supplement: Supplementary file 1 — Supplementary Material 1: Figure S1. Variations in microvillar height and pitch among distinct thin sections (I–V). A, Physalia physalis. B, Velella velella. Numbers in parentheses indicate the number of measurements within each section. [file 40851_2025_253_MOESM1_ESM.pdf]

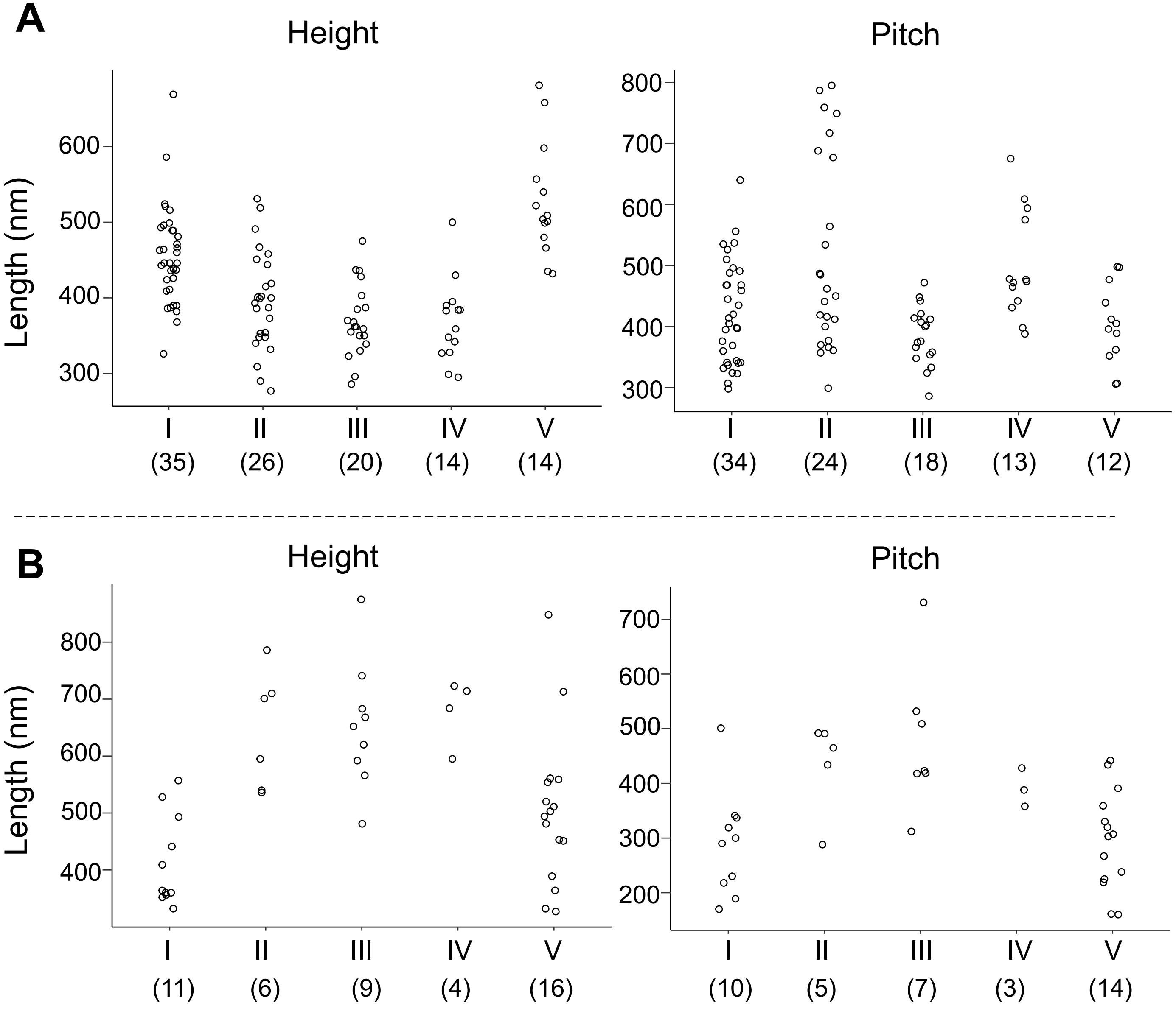

Figure S1. Variations in microvillar height and pitch among distinct thin sections (I–V). **A**, *Physalia physalis*. **B**, *Velella velella*. Numbers in parentheses indicate the number of measurements within each section.
